# Heart Plasticity in Response to Pressure- and Volume-Overload: A Review of Findings in Compensated and Decompensated Phenotypes

**DOI:** 10.3389/fphys.2020.00092

**Published:** 2020-02-13

**Authors:** Fotios G. Pitoulis, Cesare M. Terracciano

**Affiliations:** Myocardial Function, National Heart and Lung Institute, Imperial College London, London, United Kingdom

**Keywords:** myocardial remodeling, pressure overload, volume overload, mechanical load, heart failure, concentric hypertrophy, eccentric hypertrophy, myocardial slices

## Abstract

The adult human heart has an exceptional ability to alter its phenotype to adapt to changes in environmental demand. This response involves metabolic, mechanical, electrical, and structural alterations, and is known as cardiac plasticity. Understanding the drivers of cardiac plasticity is essential for development of therapeutic agents. This is particularly important in contemporary cardiology, which uses treatments with peripheral effects (e.g., on kidneys, adrenal glands). This review focuses on the effects of different hemodynamic loads on myocardial phenotype. We examine mechanical scenarios of pressure- and volume overload, from the initial insult, to compensated, and ultimately decompensated stage. We discuss how different hemodynamic conditions occur and are underlined by distinct phenotypic and molecular changes. We complete the review by exploring how current basic cardiac research should leverage available cardiac models to study mechanical load in its different presentations.

## Introduction

The heart is a terminally differentiated organ aimed at maintaining cardiac output (CO) to the rest of the body. Changing metabolic demands require systems and signaling pathways in place to allow the heart to (a) operate across a range of contractile profiles in the short term, and (b) chronically alter its function and structure when these are of enduring nature. The latter process, whereby the heart changes its phenotype chronically, is termed cardiac remodeling or plasticity.

Cardiac plasticity is a complex and multifactorial process. It is driven by mechanical load, the neurohormonal axis (Cohn et al., 2000; Yang et al., 2016), inflammation (Anzai, 2018), as well as autocrine and paracrine mediators (Gnecchi et al., 2008). Within the context of a whole organism these remodeling actors are interconnected and directly or indirectly influence one another. A decrease in systolic blood pressure decreases systolic load, and is met with hormonal release, including angiotensin II and catecholamines. These not only modulate the function of the heart, but that of the vasculature as well, which in turn has mechanical consequences on the operation of the heart. Complex feedback loops are established in this manner, fine tuning the acute functional outputs of the heart while simultaneously driving remodeling.

There are numerous reviews (de Tombe, 1998; Burchfield et al., 2013) on pathological remodeling as seen in end-stage heart failure (HF). Here, we focus less on the terminal phenotypes and more on the process of ventricular remodeling as it occurs during pressure- and volume-overload. When the information allows it, we attempt to temporally track remodeling from initial event and its immediate effects on heart function, to changes seen in the compensated, and ultimately decompensate stage. Emphasis is placed on contractile and metabolic remodeling, and the interaction of these. The electrical apparatus is discussed vis-à-vis its capacity to induce sustained mechanical dysfunction.

### Basis for Cardiac Remodeling

Cardiac remodeling is adaptive and has evolutionary underpinnings. A fight-or-flight event elicits acute physiological responses, such as β-adrenergic activation, to increase CO. These are beneficial in the short-term as they take us away from danger; yet persistent stressor exposure drives maladaptive remodeling leading to decompensation (Swynghedauw, 2006; Hill and Olson, 2008). Still, the ability to remodel is evolutionary advantageous (Shave et al., 2019). When compared to sister taxa species (e.g., chimpanzees), human hearts are comparatively longer, thinner, and less trabeculated and so optimized to maintain CO during low-to-moderate intensity endurance activities (e.g., farming) (Shave et al., 2019). Such phenotypes are not fail-proof and when short bursts of resistance-dominated activities (fight, lift, flight) are needed, failure to increase CO can have dire consequences for survival (Shave et al., 2019). As such, conservation of cardiac phenotypic plasticity permits stimuli-driven cardiac adaptations and survival.

Changes in heart structure and function of rowers and skiers were recognized by pioneering investigators as early as late 19th century, and the concept of exercise-induced cardiac remodeling is now widely accepted (Weiner and Baggish, 2012). Likewise, left ventricular mass increases during pregnancy, a physiological state of chronic volume-overload, but regresses to normal levels post-partum (Mesa et al., 1999; Hill and Olson, 2008). It is hypothesized that under physiological stimulation the heart remodels without subsequent decompensation. In contrast, arterial hypertension, myocardial infarction, and valvular disease lead to cardiac hypertrophy, albeit in different forms, which if not corrected progresses to overt decompensation. At least in the initial phases, phenotypic overlap exists between physiological and pathological remodeling (Hill and Olson, 2008). Binary categorization may be too simplistic and it is more likely that remodeling occurs across a continuum (Dorn, 2007). This is supported by clinical and pre-clinical observations of not only progressive pathological- but also reverse-remodeling (Gheorghiade et al., 2016), as seen for example with implantation of mechanical assist devices (Ibrahim and Terracciano, 2013; Kormos et al., 2017) or when the cause of adverse remodeling is corrected (Lamb et al., 2002).

## Pressure-Overload

**FIGURE 1:**
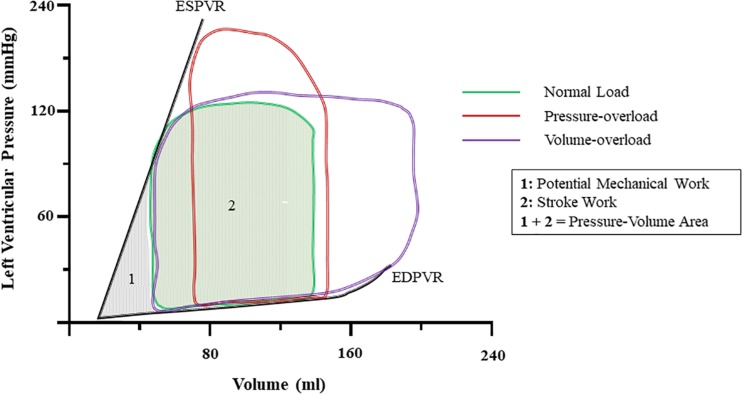
Ventricular pressure-volume dynamics under normal and at onset of pressure-, and volume-overload. Notice that increases in pressure increase left-ventricular pressure and abbreviate stroke volume. Volume-overload shifts the EDV to the right, and SV increases due to higher preload. The sum of the area encompassed by the PV-loop of normal (Cohn et al., 2000) and the ESPVR and EDPVR (Yang et al., 2016) is known as pressure-volume area (PVA) and it correlates with myocardial oxygen consumption. ESPVR, end-systolic pressure-volume relationship; EDPVR, end-diastolic pressure-volume relationship; SV, stroke volume; EDV, end-diastolic volume.

An increase in arterial blood pressure represents an increase in afterload. Cardiac physiology dictates that for the same inotropic state, a reduction in stroke volume (SV), and by extension CO ensues (Figure 1). Compensatory release of catecholamines, among other hormones as well as intrinsic myocardial responses, increase contractility to preserve CO. However, persistent exposure to growth factors results in hypertrophic cardiac growth. If left uncorrected, contractile, electrophysiological, metabolic, and structural abnormalities occur. Together, these orchestrate the progressive decline in cardiac pump function (Schirone et al., 2017).

### Cardiac Hypertrophy

**FIGURE 2:**
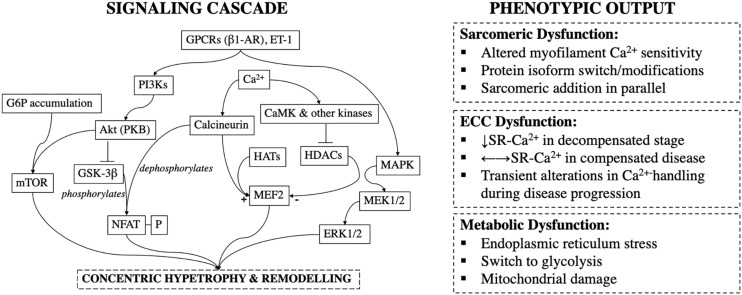
Signaling cascade and phenotypic outcomes during pressure-overload induced cardiac remodeling; GPCRs, G-Protein Couple Receptors; β1-AR, β1-Adrenoceptor; ET-1, Endothelin-1; PI3Ks, Phosphoinositide-3-Kinases; Akt (PKB), Akt/Protein Kinase B; G6P, Glucose-6-Phosphate; GSK-3β, Glycogen Synthase Kinase-3β; mTOR, Mammalian Target of Rapamycin; NFAT-P, Phosphorylated Nuclear Factor Activated T-cells; CaMK, Calmodulin Kinase; HATs, Histone Acetylases; HDACs, Histone Deacetylases; MEF2, Myocyte Enhancer Factor-2; MAPK, Mitogen Activated Protein Kinase; SR, Sarcoplasmic Reticulum.

**FIGURE 3:**
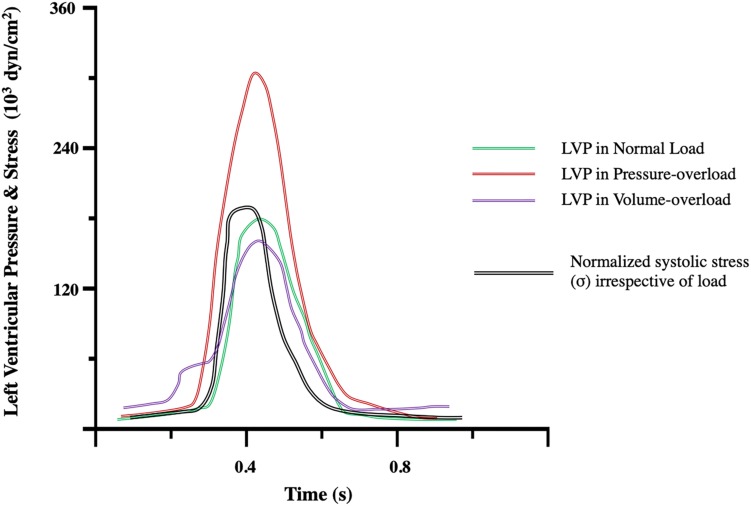
Left ventricular pressure (LVP) and stress (σ) transients during the compensatory phase of pressure-, and volume-overload and compared to normal load. Notice that hemodynamic profiles differ in terms of cardiac LVP generation. The ventricular chamber must generate pressure greater than that in the aorta to eject blood. An increase in afterload in pressure-overload results in increased LVP. In the compensated state, stress is normalized by thickening of the ventricular wall according to LaPlace’s law. Modified from Figure 5 of Grossman et al. (1975).

Sustained elevated systolic stress via thoracic aortic constriction (TAC) in mice, increases the rate of protein synthesis as soon as 4 days post-op and results in cardiac hypertrophy (Wang et al., 2017) (Figure 2). The putative hypothesis of stress normalization, proposed by Grossman et al. (1975), argues that elevations in systolic stress are offset by an increase in wall thickness. This results in normalization of stress according to LaPlace’s law^1^ (Figure 3). In pressure-overload, it is generally accepted that ventricular wall thickening occurs by growth of cardiomyocytes via addition of sarcomeres in parallel within myofibrils. At the level of the whole heart, this leads to concentric hypertrophy, and chamber geometry of increased ventricular wall-thickness *(h)* to radius *(r)* ratio (Grossman and Paulus, 2013). However, this hypothesis has been questioned. Rat right ventricular papillary muscles cultured under isometric- (supraphysiological systolic stress) or isometric-load with the contraction uncoupler BDM (reduced systolic stress) both develop increased cardiomyocyte diameter and decreased length in comparison to physiologically loaded muscle, suggestive of parallel rearrangement of sarcomeres (Guterl et al., 2007). As very high and very low systolic stresses both lead to similar sarcomeric rearrangement, muscle shortening profiles (rate and amount of muscle shortening or equally changes in volume; e.g., isometric twitches do not shorten) have been proposed to be more decisive than stress in driving the hypertrophic response (Guterl et al., 2007). More importantly, irrespective of the driver of wall thickening it is still unclear whether this initial compensatory phase is required and whether it is in fact beneficial.

Cardiac hypertrophy is an independent and the single most important risk factor for cardiovascular morbidity and mortality (Manyari et al., 1990; Balke, 1998). In TAC-induced pressure-overloaded mice, inhibition of calcineurin, a hypertrophic signal transduction molecule (Figure 2), abolishes development of compensatory cardiac hypertrophy without deterioration of LV function and structure, or progression to HF (Hill et al., 2000).

Other studies show that hypertrophic adaptations are necessary. Stromal interaction molecule 1 (STIM1) is a regulator of cardiomyocyte growth (Schirone et al., 2017). In pressure-overload, STIM1 silencing prevents cardiac hypertrophy but results in rapid deterioration to HF (Benard et al., 2016; Schirone et al., 2017). Moreover, studies on isolated cardiomyocytes from patients on mechanical assist devices show that regression of hypertrophy *per se* is not associated with clinical recovery (Terracciano et al., 2004). Instead, both patients who recover and those who do not recover show decreased cell size but the former demonstrate renormalization of excitation-contraction features, such as sarcoplasmic reticulum Ca^2+^-content, and L-type Ca^2+^ current (Terracciano et al., 2004). More, concentric hypertrophy is a physiological response to strength exercise, where activation of vasopressor systems and increases in peripheral resistance can elevate the aortic pressure up to 320/225 mmHg (Mihl et al., 2008).

Such polarizing findings muddle our understanding of the protective and/or detrimental effects of hypertrophy. Part of the explanation for this stems from incomplete mapping of responsible pathways. Not all hypertrophic pathways are maladaptive. For example, mice dominant-negative for phosphoinositide 3-kinase (PI3Ks) p110α-isoform, develop hypertrophy after aortic constriction but not exercise (McMullen et al., 2003) (Figure 2). This suggests that in contrast to harmful effects of calcineurin activation (Sussman et al., 1998), PI3Ks may be cardioprotective and promote beneficial adaptations to stress (McMullen et al., 2003; Crozatier and Ventura-Clapier, 2015). However, others have reported that calcineurin mediates physiological responses (Wilkins and Molkentin, 2002). It is likely that overlap exists in signaling pathways of physiological, adaptive, and pathological reactive hypertrophy (Dorn, 2007), and sharp distinctions may not be possible. Clinically, this perturbs the therapeutic line of what we can and cannot safely manipulate. Inhibition of protective or sustained elevation of pathological pathways may lead to the same net deleterious outcome.

### Contractile Remodeling

#### Sarcomeric Dysfunction

Sarcomeric protein isoform expression and post-translational modifications are key determinants of sarcomeric function (Yin et al., 2015). The healthy human adult heart is β-myosin heavy chain (MHC) dominant (Miyata et al., 2000), with α-MHC density suggested to range from less than 10% (Taegtmeyer et al., 2010) up to 30% (Hamdani et al., 2008). A switch from α- to β-MHC has been reported during pressure-overload (Crozatier and Ventura-Clapier, 2015) but whether this is happening in humans remains controversial (de Tombe, 1998). β-MHC is more energetically efficient in developing force, whereas α-MHC has faster tension kinetics and power output (Herron and McDonald, 2002; Taegtmeyer et al., 2010). Isoform switch may thus be beneficial in the face of increased oxygen demand due to elevated systolic wall stress, but at the expense of contractile speed (Hamdani et al., 2008; Stelzer et al., 2008). As twitch duration impacts SV, α-MHC may be better equipped to maintain CO when the heart is overloaded. Consequently, the heart may struggle to cope with workload following isoform switch and this may contribute to pathological remodeling in a positive-feedback fashion.

If isoform switch does happen, the magnitude of switch appears related to the originally dominant isoform; in the healthy rat heart, where α-MHC expression is high, the shift to β-MHC can be quantitatively bigger than in the human heart (Schwartz et al., 1992). This is particularly relevant given that MHC isoform composition varies within the ventricular wall. α-MHC shows a transmural gradient highest in subepicardial than subendocardial fibers (Stelzer et al., 2008). Transmural heterogeneity in other sarcomeric proteins and their post-translational status, such regulatory light chain phosphorylation (RLC-p), has also been reported (Davis et al., 2001). In healthy hearts, intricate protein isoform and phosphorylation patterns across the wall together with electrical heterogeneity (Cordeiro et al., 2004) facilitate efficient mechanical performance by optimizing the complex three-dimensional twisting and compressing motion of the whole heart (Davis et al., 2001). Yet, transmural protein profiles create margins for these to be regionally disrupted. This could lead to mechano-electrical mismatch across the ventricular wall and contribute to disease development and progression. As thorough assessment of transmural mechanical properties has not been yet performed, the consequences of this warrant further study.

Changes in myofilament Ca^2+^ sensitivity have been reported in TAC-induced hypertrophy and linked to systolic dysfunction (Belin et al., 2006). However, systolic dysfunction is not typically evident during the initial compensatory stages of remodeling to pressure-overload (Franklin et al., 1975; de Tombe, 1998) and the temporal progression of changes in myofilament Ca^2+^ sensitivity have only recently been illuminated. Rupert et al. examined ventricular function and structure in relation to sarcomeric function after 6, 12, and 16 weeks of TAC in rats. At 6-weeks post-op, myofilament Ca^2+^-sensitivity was significantly increased, whereas it normalized back to sham-group levels at 12- and 18-weeks post-op. These findings correlated temporally with an increased ESPVR at 6-weeks post-op, suggesting increased inotropic state (Suga et al., 1973; Ruppert et al., 2019). Early compensatory response to pressure-overload may therefore be predicated upon favorable adaptations in myofilament Ca^2+^-sensitivity, which are eventually lost in later stages of hypertrophy. Likewise, the Frank-Starling mechanism – that is, an increase in contractile output per unit of preload, seems to be preserved in compensated disease. In isolated papillary muscles from rats with compensated ventricular hypertrophy, maximum developed active tension is preserved (Okoshi et al., 2004). Similarly, in spontaneously hypertensive rats with ventricular hypertrophy, cardiac performance is maintained at 11- and 24-weeks when assessed with *in vivo* preload stress (by venous infusion), but falls sharply at 83-weeks of age (Pfeffer et al., 1979). Whether the Frank-Starling response is preserved at later stages of disease remains an open question.

In human hearts, Schwinger et al. (1994) suggested that the terminally failing heart is unable to employ the Frank-Starling mechanism due to loss of length-dependent activation. Exhaustion of the Frank-Starling reserve has similarly been reported in dogs with congestive HF (Komamura et al., 1993) and spontaneously hypertensive rats (Hallbäck et al., 1975). More, in right-ventricular pressure-overload hypertrophy that has progressed to decompensation, tension development in skinned isolated myocytes is reduced (Fan et al., 1997). As hypertrophy can impact the content of ECM, these experiments suggest that intrinsic changes in contractile protein function may underlie the altered contractile phenotype. However, others have shown preserved Frank-Starling in end-stage HF (Holubarsch et al., 1996; Pieske et al., 1997; Weil et al., 1998) with a leftward shift in the force-pCa curve suggesting increased Ca^2+^ sensitivity at higher sarcomere lengths (preload), and thus maintained length-dependent activation (Holubarsch et al., 1996).

As mentioned herein, pressure-overload induces concentric hypertrophy, reflected by parallel addition of sarcomeres at the level of the cardiomyocyte. Given the simultaneous incessant motion of the heart, mediated by sarcomeric shortening, and cyclic stresses, it is remarkable how new elements can be added without disruption of active cardiac mechanics. Live imaging of neonatal rat cardiomyocytes illustrates that upon stretch, parallel sarcomeric addition occurs by using pre-existing sarcomeres as templates (Yang et al., 2016). It is hypothesized that resident sarcomeres act as templates and allow for the sequential addition of “Z-bodies” that initially look like beads (Sanger et al., 2010) and progressively build up to form linear Z-disks (Yang et al., 2016). Subsequently, assembly and introduction of sarcomeric proteins (e.g., myosin, actin, titin, etc.), and the formation of new sarcomeres takes place (Russell et al., 2010; Yang et al., 2016) leading to the concentric hypertrophic geometry.

#### Ca^2+^ Handling

In decompensated disease with systolic dysfunction, there is decreased ability to sequester Ca^2+^ in the sarcoplasmic reticulum (SR) and/or release it (de Tombe, 1998). Ca^2+^-uptake in the SR is carried out by the SR Ca^2+^-ATPase (SERCA). Reductions in SERCA density and/or its Ca^2+^ uptake rates (De la Bastie et al., 1990; Sharma et al., 1994; de Tombe, 1998) can thus compromise SR Ca^2+^-availability. This can diminish the magnitude of the next and the relaxation kinetics of the preceding contraction.

There is an arsenal of evidence that this is happening (Limas et al., 1987; Hasenfuss et al., 1990; Beuckelmann et al., 1995; Flesch et al., 1996). However, most studies have been performed in dilated cardiomyopathy (Limas et al., 1987; Hasenfuss et al., 1990; Flesch et al., 1996) or end-stage HF (Beuckelmann et al., 1995; Flesch et al., 1996) samples. What is less clear is the progressive change in SERCA density and function following initial trigger. Guinea-pigs with compensated cardiac phenotype (increased LV mass with normal function) 4- and 8-weeks post-TAC, do not show changes in SERCA protein expression or function whereas animals with decompensated phenotypes (increased LV mass with depressed function) do (Kiss et al., 1995). Coupled with increased myofilament Ca^2+^ sensitivity preserved SERCA function may uphold ventricular performance during the early compensated phases of remodeling.

SERCA activity is tonically inhibited by phospholamban (PLB). PLB phosphorylation by cAMP- or Ca^2+^/calmodulin-dependent (CaMKII) protein kinases, lifts the inhibition and the SR Ca^2+^ uptake rate is increased (Bers, 2002). Hyperphosphorylation of PLB has been recorded in severe but compensated LV canine hypertrophy following aortic banding (Song et al., 2005). PKCα decreases PLB-phosphorylation via a pathway involving protein phosphatase-1, resulting in decreased SR-Ca^2+^ uptake, Ca^2+^ transient, and contractility (Braz et al., 2004). As such, PKCα deletion has been proposed to be cardioprotective against pressure-overload, yet mice without PKCα demonstrate cardiac hypercontractility (Braz et al., 2004). Given the potential of positive inotropic agents for long-term deleterious consequences, whether PKCα inhibition is of therapeutic benefit remains to be seen (Packer, 1993; Swynghedauw and Charlemagne, 2002). In contrast, nitroxyl (HNO), a product of nitric oxide (NO) reduction, is a positive inotropic agent that increases SR-Ca^2+^ content without raising intracellular Ca^2+^-content that may be deleterious. Recently, it was suggested that the mechanism underlying HNO-mediated SR-Ca^2+^ content increases is attained by keeping PLB in its oligomerized form (pentameric), which is less inhibitory than the monomeric PLB counterpart when associated with SERCA (Sivakumaran et al., 2013).

#### Excitation Contraction Coupling (ECC)

During calcium-induced calcium release (CICR) Ca^2+^ influx (*I*_*Ca*_) through sarcolemmal L-type Ca^2+^ channels (LTCCs) causes release of Ca^2+^ from the SR via SR Ca^2+^ release channels. Spatial proximity between LTCCs and SR Ca^2+^ release units is necessary for efficient CICR. This is accomplished by the highly organized network of T-tubules running throughout the entire cardiomyocyte. Rat isolated cardiomyocytes, examined during a period of hypertension before development of compensated hypertrophy have myocytes with longer action potential duration, Ca^2+^ transients, and increased ECC gain (that is *I*_*Ca*_ can trigger greater SR Ca^2+^ release) compared to non-hypertensive rats (Chen-Izu et al., 2007). Initial alterations in Ca^2+^ homeostasis thus seem to precede hypertrophy and may promote it (e.g., by activation of calcineurin and CaMKII) (Chen-Izu et al., 2007) (see below and Figure 2). In contrast, during the compensated phase, Ca^2+^-transient, and % shortening of unloaded cardiomyocytes is preserved but not elevated compared to control (Nagata et al., 1998). It is likely that an initiation window exists before compensatory remodeling where cardiomyocytes become hyperfunctional, driving disease progression.

ECC gain is reduced in decompensated disease (Gomez et al., 1997). Geometrical abnormalities in t-tubular architecture can reduce the coupling between LTCCs and SR Ca^2+^ release units and hamper ECC (Santana et al., 1996; Gomez et al., 1997). T-tubular system remodeling begins prior to the onset of heart failure in pressure-overload (Guo et al., 1996; Wei et al., 2010). Although the temporal details are not known, it is possible that during compensation maladaptive changes in t-tubule structure exist, but overall function is preserved as a result of increased myofilament sensitivity and/or improved Ca^2+^ handling (see above).

CaMKII is an intracellular serine/threonine protein kinase that acts as a Ca^2+^-dependent hypertrophic pathway (Backs et al., 2009; Maier, 2012). Under basal conditions CaMKII activity is minimal because its catalytic domain is blocked by its regulatory domain (Anderson et al., 2011). Binding of calcified calmodulin (Ca-CaM) to the regulatory domain frees the catalytic site, increasing CaMKII activity (Anderson et al., 2011). Pharmacological inhibition (Zhang et al., 2005) and genetic knockouts (Ling et al., 2009) of CaMKII, prevent progression of pressure-overload to pathological remodeling. Spontaneous diastolic Ca^2+^-leak from SR Ca^2+^-release channels (Ca^2+^-sparks) can regulate CaMKII activity (Heineke and Molkentin, 2006; Van Oort et al., 2010). In compensated LV hypertrophy increased Ca^2+^-sparks have been reported (Song et al., 2005) and TAC pressure-overload causes increased Ca^2+^ spark frequency associated with CaMKII activation (Toischer et al., 2010). The hypertrophic response in mice with leaky RyR2 channels (R176Q knock-in mutation), is accelerated and there is propensity for progression to dilated cardiomyopathy compared to wild-type (Van Oort et al., 2010). These findings are in line with clinical evidence showing that genetically defective RyRs are associated with hypertrophic cardiomyopathy predisposition (Fujino et al., 2006; Chiu et al., 2007).

CaMKII also regulates histone deacetylases (HDACs), which bind to specific transcription factors such as myocyte enhancer factor 2 (MEF-2) to control hypertrophic gene pathways (Hulot et al., 2011). HDACs are post-translational modifying enzymes that deacetylate histones and repress transcription. In contrast, histone acetylases (HATs) acetylate histones and promote transcriptional activation; HDACs and HATs have antagonistic effects. Phosphorylation of HDACs by CaMKII impairs their association with MEF-2 and other transcription factors and enhances transcriptional activation (i.e., de-represses) (Frey et al., 2004) (Figure 2). At least three classes of HDACs have been identified with class I HDACs considered to be pro-hypertrophic, whereas class II & III HDACs suggested to negatively regulate the hypertrophic gene program (Kehat and Molkentin, 2010).

### Metabolic Dysfunction

Heart function is energetically demanding and requires constant supply of ATP. As ATP stock is limited to a few beats, continuous replenishment is critical (Ingwall, 2009). Typically, in the healthy adult heart, ATP supply is maintained by oxidative phosphorylation. When acute surges in energetic demand occur highly efficient glycolytic and phosphotransferase pathways are employed to boost ATP production (Ingwall, 2009). The latter involves conversion of ADP and phosphocreatine (PCr) to ATP and creatine by creatine-kinase (CK). Physiologically, high energy-demand and oxygen consumption (VO_2_) are predicted by the pressure-volume area (PVA) – that is, the area enclosed by the end-systolic and end-diastolic pressure volume relationships and systolic work (Suga et al., 1981; Nozawa et al., 1994) (Figure 1). Correspondingly, changes in inotropy, systolic work, and diastolic function all impact cardiac energetics.

Increased afterload increases cardiac work per beat, and leads to an imbalance in the contribution of glycolytic and oxidative pathways in maintaining ATP levels (Nguyen et al., 1990; Young et al., 2007; Ingwall, 2009; Taegtmeyer et al., 2010). In particular, the heart switches from a fatty-acid dominant metabolism to the use of glucose as a primary source of energy, associated with an increase in the rate of glucose uptake (Kundu et al., 2015).

Because glucose uptake rate can exceed rate of glucose utilization, particularly within the overloaded myocardium (Nguyen et al., 1990), glucose accumulation, in the form of glucose-6-phosphate (G6P) occurs (Kundu et al., 2015). This precedes the development of hypertrophy and has been reported to occur as early as 1-day post-TAC surgery (Kundu et al., 2015), and before LVH in spontaneously hypertensive rats (Hernandez et al., 2013). G6P accumulation contributes to pathological progression in two ways. First, it can activate the mechanistic target of rapamycin complex 1 (mTORC1), a major pathway of cardiac remodeling (Proud, 2004) (Figure 2). Sustained mTORC1 activation induces protein synthesis rates that cannot be matched by the endoplasmic reticulum’s (ER) protein folding capacity. This can lead to ER stress, misfolded proteins and ultimately cell death and diminished contractile output (Proud, 2004; Glembotski, 2008). Secondly, high G6P levels may directly activate the fetal gene program, by activation of the hexosamine biosynthetic pathway and subsequent glycosylation of transcription factors (Young et al., 2007). Metabolic shifts can thus precede, trigger, and maintain the reinduction to the fetal gene program (Taegtmeyer et al., 2010). In TAC-rats, low-carbohydrate/high-fat diet minimizes α- to β-MHC isoform switch (Young et al., 2007). Furthermore treatment with propranolol decreases glucose uptake rates, G6P accumulation, and rescues the decrease in ejection fraction observed in vehicle-treated rats (Zhong et al., 2013).

The consequences of a fetal energetic state are reductions in [ATP] by as much as 30% in diseased compared to healthy hearts (Ingwall, 2009). Because ATP is necessary for normal contraction, reduced ATP levels means that the failing heart is energetically starved. Until recently it was unclear whether this was one of the many failing phenotypic consequences or contributes directly to disease progression (Gupta et al., 2012). In TAC pressure-overloaded mice, overexpression of creatinine kinase-M (CK-M) improves LV contractile function and augments contractile reserve compared to TAC-controls. Importantly, these functional effects are lost when CK-M overexpression ceases (Gupta et al., 2012). These findings suggest that energy starvation, in this case attributed to CK levels and activity, can directly contribute to mechanical dysfunction and the development of disease.

Pressure-overload also causes mitochondrial damage. This leads to mitochondrial dysfunction, disturbances in substrate utilization and mitochondrial respiration, a decrease of fatty acids and an increase of glucose oxidizing proteins (Dai et al., 2012). The majority of proteins are imported into mitochondria, where they undergo folding and assembly (Smyrnias et al., 2019). Production of reactive-oxygen species in pressure-overload (ROS) (Goh et al., 2019) promotes mitochondrial protein misfolding, dysfunction, and a perpetual cycle of progressively worsening mitochondrial damage (Smyrnias et al., 2019). A major source of ROS production are monoamine oxidases (MAOs), and in particular MAO-A (Kaludercic et al., 2014). MAOs are mitochondrial flavoenzymes that catabolize neurotransmitters, such as norepinephrine and epinephrine. H_2_O_2_ is produced during this catalytic pathway resulting in oxidative stress (Kaludercic et al., 2010). In TAC, MAO-A expression and activity increases causing oxidative stress and adverse chamber dilatation (Kaludercic et al., 2010). Remarkably, mice dominant negative for MAO-A exposed to TAC, despite a slight shift in the PV relationship, have preserved cardiac function 9 weeks post TAC (Kaludercic et al., 2010). Protein misfolding due to ROS may also contribute to activation of the mitochondrial unfolded protein response (UPR^*mt*^) pathway (Smyrnias et al., 2019). This is an evolutionary conserved pathway activated in response to compromised mitochondrial folding capacity. Pharmacological enhancement of UPR^*mt*^ ameliorates mitochondrial dysfunction (Smyrnias et al., 2019).

Last but not least, compromised mitochondria can directly impair sarcomeric function and regulation. In adult cardiomyocytes, mitochondria are located close to sarcomeres in what are considered ‘energetic couplons’ (Saks et al., 2001), units primed for optimal ATP exchange. Impaired mitochondrial energetic output may constrain sarcomeric function and encourage adaptive sarcomeric responses such as isoform switch to energetically favorable β-MHC. Increases in cardiomyocyte volume during hypertrophy can also decouple these units and impose energetic diffusional barriers (Crozatier and Ventura-Clapier, 2015) further facilitating sarcomeric remodeling. Yet, the extent of the latter is not clear, as simultaneous mitochondrial biogenesis has been reported to occur during parallel sarcomeric addition (Yang et al., 2016).

### Debrief

(1) Pressure-overload causes concentric hypertrophy. This can happen in response to physiological and pathological triggers. Within the context of disease, whether hypertrophy confers protection or is deleterious remains elusive.

(2) An initial window characterized by functional and structural changes exists before overt compensated hypertrophy. Changes that occur during this window may directly contribute to the initiation of hypertrophy. In particular:

(i) Increased energetic demand is offset by changes in sarcomeric protein composition (economical isoform switches), and changes in utilization of energetic substrates (fatty acid to glucose). It is possible that energetic-contractile signaling exists, mediated (a) directly due to proximity of energy-producing and energy-consuming units, and (b) indirectly by glycosylation of transcription factors that regulate sarcomeric apparatus.
(ii) Changes in ECC function and structure such as Ca^2+^-handling precede development of hypertrophy and initiate it (e.g., via Ca^2+^-dependent hypertrophic pathways).

(3) Compensation does not preclude maladaptive remodeling. T-tubular loss, mitochondrial dysfunction, and sarcomeric dysfunction may all be present but masked by countering hyperfunctional cardiomyocyte elements, such as increased myofilament Ca^2+^ sensitivity. If the trigger is not corrected, the balance tilts, loss of hyperfunction occurs, and this is manifested macroscopically as overt decompensation.

## Volume-Overload

End-diastolic volume corresponds to ventricular preload. In volume-overload, such as aortic and mitral valve insufficiency and certain congenital abnormalities, excessive preload is imposed on the heart. For example, in mitral valve regurgitation, following LV ejection a volume of blood is displaced back into the lower-pressure left atrial chamber; ventricular filling of the next contraction is increased causing volume-overload. In such settings, patterns of ventricular remodeling can be very different to pressure-overload.

Eccentric hypertrophy is the hallmark of volume-overload, whereby the chamber dilates while wall thickness decreases or is maintained. Wall thickness is particularly important. As a compensatory response, eccentric hypertrophy develops to preserve SV in the face of excess volume (Carabello et al., 1992). According to LaPlace’s law chamber enlargement would lead to acute increases in systolic stress (caused by decreased *h/r* ratio) that would be subsequently normalized by thickening of the LV wall so that mass-to-volume ratio is preserved (Grossman et al., 1975; Opie et al., 2006) (Figure 3). However, although LV mass has been reported to increase (Grossman et al., 1975), many have found a decrease in mass-to-volume ratio (Corin et al., 1987; Carabello et al., 1992; de Giovanni, 2004), and so uncompensated stress leading to maladaptive remodeling (Carabello et al., 1992; Toischer et al., 2010). In contrast, physiological eccentric hypertrophy occurs in endurance athletes where LV mass closely mirrors increases in volume (Hoogsteen et al., 2003; Mihl et al., 2008).

During progression to heart failure from compensated pressure-overload, significant ventricular dilatation is observed resembling eccentric geometry (Randhawa and Singal, 1992). This is different from the eccentric hypertrophy seen during the compensated phases of volume-overload, where SV is maintained despite increased chamber size (Carabello, 2002).

### Contractile Remodeling

#### Sarcomeres and Cardiomyocyte Morphology

The initiating trigger for eccentric hypertrophy is postulated to be excess end-diastolic wall stress or preload (Grossman et al., 1975; Urabe et al., 1992). Preload is the mechanical stretch and tension encountered by the myocardium during the diastolic phase. It represents the passive component of twitch tension, or passive tension. High preload causes cardiomyocytes to lengthen and thin as sarcomeric apparatus is rearranged and sarcomeres are added in series (Kehat and Molkentin, 2010).

**FIGURE 4:**
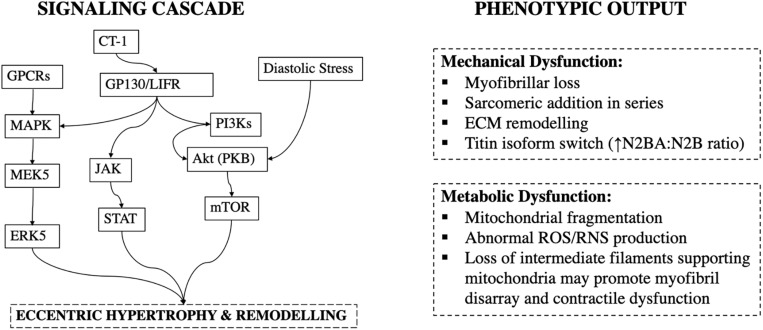
Signaling cascade and phenotypic output in volume-overload. CT-1, Cardiotrophin-1; GP130/LIFR, Glycoprotein-130/Leukemia Inhibitory Factor Receptor; JAK, Janus Kinases; STAT, Signal Transducer and Activator of Transcription Proteins.

This type of sarcomeric remodeling is mediated by specific signaling pathways. Gp130 is a transmembrane protein of cytokine receptors and a critical component for signal transduction following cytokine engagement (Giese et al., 2005). Cardiotrophin-1, a cytokine, has been shown to cause gp130 homodimerisation or heterodimerization with leukemia inhibitory factor receptor β (LIFRβ) and trigger sarcomeric addition in series, and cardiomyocyte lengthening in volume-overload (Wollert et al., 1996) (Figure 4).

Optimal force generation occurs across a narrow spectrum of SLs (Hibberd and Jewell, 1982; Allen and Kentish, 1985) meaning that resting SL must be maintained (Urabe et al., 1992; Mansour et al., 2004). This ensures that when preload increases and cardiomyocytes are stretched, the SL-force relationship is preserved. Accordingly, though cardiomyocytes elongate under increased diastolic stress, their resting SL remains normal, reflecting sarcomeric addition in series (Gerdes et al., 1992). Uniaxial strain in isolated neonatal rat cardiomyocytes causes an acute ∼6% increase in SL, which however renormalizes back to resting SL after 6 h in culture (Mansour et al., 2004). The mechanical sensors that orchestrate this are still unclear. Protein kinase C ε-subtype (PKCε) and focal adhesion kinase (FAK) have been reported (Mansour et al., 2004). These colocalize in costameres and cardiomyocyte focal adhesions, where they sense and mediate initiation of sarcomerogenesis in response to changes in stress and strain (Russell et al., 2010). Myofibril elongation is then suggested to take place by two mechanisms.

Firstly, insertion of new sarcomeres close to the cell-membranes, and most likely at intercalated disks (ID) (Wilson et al., 2014). IDs are complex folded structures where adjacent cardiomyocytes come in contact. Adherens junctions at the ID connect to the terminal thin filaments of sarcomeres sensing tension (Wilson et al., 2014). A special region within IDs known as transitional junction, can act as a “proto Z-disk” where new sarcomeres can be added (Yoshida et al., 2010). The second mechanism is insertion within an existing myofibril via splitting. This occurs when a myofibril splits open to allow for insertion of a new sarcomere at the cleaved site (Yang et al., 2016).

Myocyte elongation may put them at a mechanical disadvantage when generating force (Gerdes and Capasso, 1995). It is possible that in eccentric remodeling the same amount of preload causes a different SL change; put simply, longer cardiomyocytes may need to be stretched more, compared to their normal counterparts, to reach a given SL. Thus, although the SL-force relationship may be preserved, force production may be compromised because myocardial tissue would require a greater (and perhaps supraphysiological) preload to reach the same SL.

#### Mechanical Dysfunction

In volume-overload from surgically induced aorto-caval shunts in rats, isoform switch from α- to β-MHC has been noted (Guggilam et al., 2013). Isolated cardiomyocytes from these rats demonstrate impaired contractile function with reduced amplitude and velocity of shortening (Guggilam et al., 2013). However, these experiments carried out 21-week post-op may reflect end-stage HF and may occur irrespective of the original volume-overload event. For example, others have not reported isoform switch in a similar experimental model of volume-overload at 24 h (Toischer et al., 2010). Without temporal tracking of functional and molecular changes during compensated and decompensated stages it is difficult to attribute observations to volume-overload or simply a common decompensated phenotype that has originated from volume-overload. Furthermore, no differences in shortening amplitude, velocity, and relaxation of isolated cardiomyocytes 4–15 weeks (Ryan et al., 2007) and 7-days post (Toischer et al., 2010) aorto-caval shunt surgery compared to control have been reported. Myofibrillar loss has been shown to occur extensively in volume-overload in both experimental mitral valve regurgitation (Urabe et al., 1992), and in biopsies from patients undergoing repair surgery (Ahmed et al., 2010; Gladden et al., 2011) and may contribute to mechanical dysfunction.

The extracellular matrix (ECM) regulates contractile performance via direct manipulation of myocardial stiffness as well as by coordinating myocardial remodeling (Baharvand et al., 2005). In volume-overload, ECM is degraded with reductions in collagen fractional volume, and increases in elastin (Ryan et al., 2007; Wilson and Lucchesi, 2014; Hutchinson et al., 2015). Collagen reductions occur due to increased matrix metalloproteinases (MMPs) activity and a concurrent decrease in their inhibitors, tissue inhibitors of MMPs (TIMPs) (Levick et al., 2008; Wilson and Lucchesi, 2014). This is in stark contrast to pressure-overload, whereby increased collage synthesis occurs (Bishop et al., 1994) and is associated with perivascular fibrosis (Toischer et al., 2010). Elastin is more compliant than collagen and decreased elastin-to-collagen ratio contributes to arterial stiffening with age (Fomovsky et al., 2010); therefore, an increase in elastin coupled with collagen reduction may lead to a more compliant ventricle in volume-overload.

In addition to collagen, passive myocardial mechanical properties are regulated by titin. Titin is a giant protein that forms part of the sarcomeric apparatus, and within the adult heart is typically found in either a longer more compliant N2BA or a shorter and stiffer N2B isoform (Lewinter and Granzier, 2010). The N2BA:N2B ratio increases after 4-weeks of volume-overload by aorto-caval shunt in rats, and this corresponds functionally to an increase in the passive tension of skinned myofibers (Hutchinson et al., 2015) (Figure 4). This is hypothesized to be a beneficial adaptation, as increased stiffness may limit excessive eccentric remodeling (Hutchinson et al., 2015).

### Metabolic Remodeling

In volume-overload, elevated LV end-diastolic stress increases cardiac work, myocardial oxygen and ATP demand, haemodynamically reflected by a greater PVA (Suga et al., 1981; Gladden et al., 2011). Disruptions in mitochondrial function can impair energy generation and contribute to pathological remodeling. Indeed, mitochondria fragmentation has been observed in volume-overload, and this has been linked with bioenergetic deficit (Gladden et al., 2011).

One implicated pathway has been abnormal generation of reactive oxygen and nitrogen species (RNS). Mitochondria are major producers and consumers of ROS/RNS due to their high content in reactive proteins (Gutierrez et al., 2006). ROS/RNS reactions damage mitochondrial DNA, resulting in further ROS production and harmful positive feedback loops (Gladden et al., 2011; Yancey et al., 2015). Xanthine oxidase (XO) activity, involved in ROS production, is increased in the LV within the first 24 h in rats following aorto-caval shunt surgery (Gladden et al., 2011). This is accompanied by a decrease in mitochondrial oxidative function (Gladden et al., 2011), and suggests early metabolic abnormalities in volume-overload.

Mitochondrial dysfunction has also been linked to ECM remodeling (Ulasova et al., 2011). 24 h after aorto-caval shunt there is disruption of subsarcolemmal mitochondria structure, and interstitial collagen decrease, attributed to increased MMP activity (Ulasova et al., 2011). These abnormalities are corrected by pharmacological MMP inhibition (+PD166793) and mitochondrial respiration is significantly increased (Ulasova et al., 2011).

Emerging evidence also shows that increased MMP activity can have intracellular consequences, namely myofibril proteolysis (Kandasamy et al., 2010). For example, MMP-2 increases troponin-I, and myosin light chain 1 degradation in ischemia-reperfusion injury (Sawicki et al., 2005). Moreover, MMP activation is speculated to digest intermediate filaments supporting intermyofibrillar mitochondria (Ulasova et al., 2011). It is possible that this leads to contractile dysfunction by promoting myofibril disarray and loss, while disrupting the energetic coupling between mitochondria and sarcomeres.

More, similar to pressure-overload, glycemic control may impact development and progression of volume-overload by activation of hypertrophic pathways. In rats with aortic-regurgitation, high fructose intake results in worsening of eccentric remodeling (Bouchard-Thomassin et al., 2011). In pressure-overload activation of the Akt/mTOR signaling cascade is enhanced with hyperglycemia (Hernandez et al., 2013), but this pathway was not found to be upregulated in high-fructose aortic regurgitation (Bouchard-Thomassin et al., 2011). However, aortic-regurgitation does not produce pure volume-overload (Wilson and Lucchesi, 2014), and others have reported that activation of Akt/mTORC1 pathway has a central role in promoting eccentric hypertrophy in aorto-caval shunts with the level of pathway activation being a function of LV end-diastolic stress (Ikeda et al., 2015) (Figure 4).

### Debrief

(1) Volume-overload results in eccentric hypertrophy. The decreased *h/r* ratio elevates stress and is normalized by increases in wall thickness; the chamber size remains elongated. Pattern of remodeling is generally agreed to be driven by diastolic and not systolic stress.

(2) Sarcomeric addition occurs in series. This allows SL to be restored back to normal resting length following acute diastolic stretch. Though the force-SL relationship may be maintained, greater amount of stretch may be required to reach a given SL in elongated cardiomyocytes, which may impair the Frank-Starling response.

(3) Although the extent of isoform switches remains unclear, myofibrillar loss is a common feature of volume-overload, diminishing contractile performance and disrupting energetic couplons (mitochondria-sarcomeres). Mitochondrial oxidative stress may also directly cause myofibrillar loss, disrupt energetic coupling, and establish damaging feedback loops.

## Convergence and Divergence in Hemodynamic Overload

Volume and pressure-overload apply distinct hemodynamic load profiles (Figures 1, 3) and molecular responses (Figures 2, 4) on the myocardium. Generally, these are characterized by increased end-diastolic and end-systolic stress respectively. Myocardial adaptions then follow in line with intrinsic phenotypic plasticity. However, are these isolated and distinct responses and if not, is there overlap?

**FIGURE 5:**
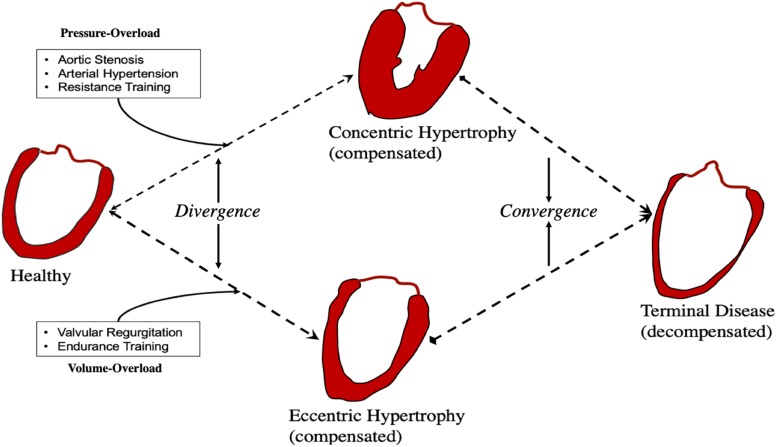
Convergence and divergence in cardiac remodeling.

If they are different responses, remodeling would diverge and lead to distinct phenotypes. In contrast, overlap would result in convergence and shared phenotypes (Figure 5).

Activation of CAMKII, and calcineurin has been shown in pressure-, but not volume-overload (Toischer et al., 2010). Likewise, titin N2BA:N2B isoform ratio is decreased in volume-overload whereas it is increased in patients with aortic stenosis (Borbély et al., 2009). In pressure-overload excess collagen deposition and fibrosis are mediated by TGF-β and contribute to mechanical dysfunction (Dobaczewski et al., 2011), whereas inhibition of TGF-β in volume-overload accelerates LV dilatation (Frantz et al., 2008; Ulasova et al., 2011). Different biochemical and gene expression profiles have been suggested to be modulated in pressure- vs. volume-overload (Miyazaki et al., 2006; Toischer et al., 2010; You et al., 2018) correspondent with different signaling pathways (Figures 2, 4). These findings support divergent pathways resulting in distinct morphological, structural, and functional myocardial properties in each load profile.

However, whether these experimental results where hemodynamic and environmental control is strictly and finely imposed are adequately representative of human pathophysiology is unclear. In patients with symptomatic aortic stenosis, eccentric hypertrophy has been noted (Villari et al., 1995). Likewise, titin N2BA:N2B ratio has been found to increase not only in compensated volume (Hutchinson et al., 2015), but also in pressure-overload (Williams et al., 2009).

It therefore seems that despite distinct signaling pathways mediating responses to load, convergence promoters must also be at play, shifting the myocardium toward common phenotypes. Identification of points of convergence and divergence is important as many of the properties of therapeutic agents for pressure-overload may not be shared in volume-overload and vice-versa (You et al., 2018). Patterns of convergence and divergence may be underlined by quantitative and qualitative agents: namely *time*, and *nature and spectrum of stimulus*.

The first obvious convergence promoter is *time*. Temporal phenotypic heterogeneity is often seen at first in cardiac disease, and as we have described herein, early adaptive phases following insult result in stark phenotypic differences in volume- and pressure-overload. Yet terminal HF phenotype share many similarities irrespective of initial trigger. It is possible that *compensated* remodeling is associated with *divergence* whereas *decompensated* remodeling with *convergence*. Clinically, this may be important as treatment during divergence may (a) be more likely to work, and (b) be more likely to be tailored to the phenotype being treated.

Secondly, the *nature and spectrum* of initiating stimulus. For example, arterial hypertension comprises a large range of volume- and pressure-load profiles (de Giovanni, 2004). In myocardial infarction there is both pressure- and volume-overload and remodeling includes both early LV dilation and hypertrophy (Opie et al., 2006). Likewise, aortic regurgitation has components of both volume- and pressure-overload (Carabello, 2002). In the presence of multiple and overlapping stimuli *convergence* will be favored. Similarly, with progression to decompensation, multiple signaling pathways that were previously quiescent now become active, and *convergence* dominates.

When initial events have distinct hemodynamic profiles, divergent phenotypes appear during the initial compensatory phases. With time, activation of multiple overlapping pathways leads to decompensation and phenotype convergence. Mixed phenotypes are possible in real-life where elements from both pressure- and volume-overload are likely to exist in sync.

## Experimental Models to Study Load-Induced Remodeling

In preclinical *in vivo* cardiac research, mechanical load cannot be studied in isolation to the neurohormonal axis. This is because, irrespective of complexity of experimental design to minimize neurohormonal activation (Swynghedauw, 2006), disrupting one inadvertently disrupts the other (Russell et al., 2010). Mechanical load and the neurohormonal axis can both drive cardiac remodeling, and can activate different gene programs (Frank et al., 2008). For example, many studies on RV volume-overload do not show deterioration of chamber contractility, yet when autonomic activity blockade is employed, RV contractile performance shows an immediate and persistent decline (Shah et al., 2000). Without model reduction, attained with utilization of simpler *in vitro* systems, pinpointing the contribution of the hormonal and mechanical axis, the activation of specific pathway mediators and their therapeutic potential is not only challenging but potentially misleading.

Although each cardiac model has unique advantages and limitations, many contemporary *in vitro* assays are oversimplified, and/or have low experimental throughput (Pitoulis F. G. et al., 2019). For example, isolated cardiomyocytes lack extracellular matrix (Watson et al., 2017). The relevance of engineered heart tissue (EHT) constructs to adult myocardium needs to be validated (Dhahri et al., 2018), and papillary muscles, whole hearts, and trabecula may suffer from low experimental throughput (Schechter et al., 2014). Additionally, appropriate protocols to simulate physiological and pathological mechanical load *in vitro* are not in widespread use. Sine-wave cyclic stretch in isolated cardiomyocytes (Yancey et al., 2015), and isotonic or isometric protocols in engineered heart tissues (Weinberger et al., 2017; Leonard et al., 2018) do not reflect the complexity of *in vivo* cardiac mechanics. Given the importance of pressure-volume and stress-strain relationships, at different times during the cardiac cycle in directing remodeling, physiology-inspired approaches are needed to simulate mechanical load *in vitro* and study its phenotypic consequences reliably.

In our lab we have pioneered the development of a novel *in vitro* cardiac model known as myocardial slices (Perbellini et al., 2017; Watson et al., 2017, 2019; Pitoulis F. et al., 2019). Myocardial slices are living organotypic preparations that can be prepared from mammalian hearts including human biopsies (Watson et al., 2017). The nature of the preparation means that myocardial functional (contractility, electrophysiology) and structural properties (ECM, hetero- and multi-cellularity) of the original tissue are preserved. Additionally, multiple slices can be prepared from a single specimen allowing for high-throughput experimentation. We and others have shown that slices can be kept in *in vitro* culture for prolonged periods of time with minimal loss of the adult myocardial phenotype (Kang et al., 2016; Fischer et al., 2019; Watson et al., 2019). As such, myocardial slices are uniquely positioned for interrogation of effects of chronic load on the myocardium away from the confounding influences of the neurohormonal arm.

## Concluding Remarks

### More Work on Progression of Disease and Volume-Overload

The past decades have seen massive strides in enhancing our understanding of the terminal myocardial phenotype. Experimentally, this may be easier to study. Less is known about the progression of disease from insult, to compensation, and then decompensation. Furthermore, despite much work on pressure-overload, our literature search supports that volume-overload induced remodeling is not as well documented.

### Differential Therapies in Phenotypic Convergence and Divergence

Experimental evidence, mostly from animal research, demonstrates divergent phenotypes in pressure- and volume-overload. However, the situation is probably more complicated in human pathology where multiple convergent pathways may be simultaneously activated. Using this framework, much of the heterogeneity in presentation during the compensatory phases of cardiac remodeling converges toward more homogeneous phenotypes in end-stage HF. Divergent phenotypes are more likely to benefit from tailored treatments, whereas convergence is more likely to benefit from one-size fits all approaches.

### In Need of Novel *in vitro* Cardiac Models to Study Mechanical Load

Advances in disease modeling and new methodologies have made the study of mechanical load possible in *in vitro* cardiac research; yet, fine manipulation of load is missing. The basic cardiac landscape is rapidly changing. The advent of new models coupled with new technologies is bound to open new research avenues. This will help uncover novel mediators of mechanical load in physiology, pathology, and at different stages of disease and will encourage their manipulation for therapeutic applications.

## Author Contributions

FP performed the literature search and wrote the manuscript. CT reviewed and edited the manuscript.

## Conflict of Interest

The authors declare that the research was conducted in the absence of any commercial or financial relationships that could be construed as a potential conflict of interest.

## Footnotes

1 Laplace’s law: σ = *r^∗^P/2h* where σ is systolic wall stress, *r* is LV cavity radius, *P* is left ventricular pressure, and *h* is LV wall thickness.
